# Intercellular adhesion promotes clonal mixing in growing bacterial populations

**DOI:** 10.1098/rsif.2018.0406

**Published:** 2018-09-19

**Authors:** Anton Kan, Ilenne Del Valle, Tim Rudge, Fernán Federici, Jim Haseloff

**Affiliations:** 1 Department of Plant Sciences, University of Cambridge, Cambridge, UK; 2 Departamento de Genética Molecular y Microbiología, Facultad de Ciencias Biológicas, Pontificia Universidad Católica de Chile, Santiago, Chile; 3 Fondo de Desarrollo de Áreas Prioritarias, Center for Genome Regulation, Millennium Institute for Integrative Systems and Synthetic Biology (MIISSB), Santiago, Chile; 4 Institute for Biological and Medical Engineering, Schools of Engineering, Biology and Medicine, Pontificia Universidad Católica de Chile, Santiago, Chile; 5 Department of Chemical and Bioprocess Engineering, School of Engineering, Pontificia Universidad Católica de Chile, Santiago, Chile

**Keywords:** synthetic biology, morphogenetic engineering, intercellular adhesion

## Abstract

Dense bacterial communities, known as biofilms, can have functional spatial organization driven by self-organizing chemical and physical interactions between cells, and their environment. In this work, we investigated intercellular adhesion, a pervasive property of bacteria in biofilms, to identify effects on the internal structure of bacterial colonies. We expressed the self-recognizing *ag43* adhesin protein in *Escherichia coli* to generate adhesion between cells, which caused aggregation in liquid culture and altered microcolony morphology on solid media. We combined the adhesive phenotype with an artificial colony patterning system based on plasmid segregation, which marked clonal lineage domains in colonies grown from single cells. Engineered *E. coli* were grown to colonies containing domains with varying adhesive properties, and investigated with microscopy, image processing and computational modelling techniques. We found that intercellular adhesion elongated the fractal-like boundary between cell lineages only when both domains within the colony were adhesive, by increasing the rotational motion during colony growth. Our work demonstrates that adhesive intercellular interactions can have significant effects on the spatial organization of bacterial populations, which can be exploited for biofilm engineering. Furthermore, our approach provides a robust platform to study the influence of intercellular interactions on spatial structure in bacterial populations.

## 1. Introduction

Surface growing microbial biofilms are highly prevalent in nature, and their study is relevant to both medical and industrial biotechnology. Biofilms are often made up of a complex community of organisms [1–3], whose interactions often lead to advantageous emergent behaviour [4–10]. Many such advantages are generated by an emergent spatial structure, which can act to improve a multitude of processes such as resource uptake [11,12], metabolic cooperation [13] and waste evacuation [14]. While some aspects of structure are due to the interactions of a biofilm with the environment [15–17], some are generated by self-organizing interactions of the bacteria themselves [18,19]. Given the recent interest in engineering synthetic microbial communities [20–22], and the importance of spatial structure in natural biofilms, it is important that we understand such self-organizing processes to engineer more sophisticated bacterial systems.

Self-organizing processes within bacterial populations are driven by a multitude of local chemical and physical interactions. Chemical interactions underpin a range of mechanisms such as mutualism and competition, and several studies have demonstrated their effects on the spatial organization of bacterial populations [23–25]. It is spatial constraint however, that is a defining aspect of surface growth, with bacteria growing in close physical proximity. Indeed, several studies have demonstrated that physical properties, such as cellular morphology, and physical interactions play an important role in the generation of emergent spatial organization within bacterial populations. For example, colonies of uniaxially growing rod-shaped cells generate fractal-like boundaries between lineages of cells [26], and in populations with mixed cell shapes, the cells are sorted spatially by shape [27]. Bacteria living in biofilms also produce an extracellular polymer matrix, and physical interactions between cells and the matrix can generate advantageous vertical growth [28]. Furthermore, biofilms may direct mechanical forces through the extracellular matrix by a process of regulated cell death, creating large-scale wrinkle structures [29]. Such studies demonstrate that physical interactions have major consequences for the properties of bacterial populations.

Adhesion is a pervasive phenomenon in bacterial biofilms, with microbes producing adhesive cell surface proteins (adhesins) [30], as well as adhesive extracellular polymers [1]. Adhesion allows bacterial cells to attach to surfaces and leads to the agglomeration of growing cells, and most studies into bacterial adhesion have focussed on biofilm initiation [31,32]. However, many adhesins are also expressed during biofilm growth [30]. Adhesive interactions play a crucial role in the morphogenesis of multicellular eukaryotic systems [33,34], suggesting that such interactions may play a role in biofilm spatial structure in addition to their role in attachment. While bacterial adhesion through an extracellular matrix has been shown to be competitively advantageous [28,35], the effects of direct intercellular adhesion have not been well explored.

Understanding self-organization in biology is a challenging task, as such processes are composed of many components interacting in a nonlinear fashion across many temporal and spatial scales. Such systems are difficult to probe, as a multitude of coupled parallel mechanisms have to be disentangled. Recent developments in synthetic biology have facilitated the engineering of reliable genetic components [36], as well as higher level circuits [37] and systems capable of generating multicellular patterns [38]. These developments allow for the creation of simplified genetic systems to isolate and observe the effects of specific interactions on emergent structure in a well-defined context [39]. Furthermore, engineering such simplified systems not only sheds light on the effects of a specific engineered interaction on spatial structure, but also simplifies the control and use of these interactions for bioengineering purposes.

Computational modelling is a crucial part of understanding complex systems [40], particularly when emergent properties are generated by the underlying components of a system. Through biophysical models of individual cells, we can probe how basic cellular properties give rise to global characteristics in greater detail than is available to the experimenter, and demonstrate which elements can be sufficient to generate a particular emergent property. In this work, we have used CellModeller v. 4.3 (http://haselofflab.github.io/CellModeller/), an open-source GPU-accelerated multicellular modelling framework [41]. CellModeller provides a physico-genetic simulation of bacterial colonies, modelling them in three dimensions, and can also be used to model intracellular chemical reactions and diffusion through the surrounding media. Bacteria are modelled as incompressible rigid capsules, which grow along their major axis and are bisected into equally sized daughter cells at division. Forces and motion within simulations are generated solely by cell growth, and cells experience viscous drag forces. CellModeller is capable of simulating up to 10^6^ cells in under a day, allowing for rapid and significant system scale.

In this work, we have engineered a bacterial system to exhibit intercellular adhesion through the recombinant expression of the *Escherichia coli* antigen 43 (*ag43*). *Ag43* is a self-recognizing bacterial autotransported cell surface-localized adhesin [42,43], which mediates adhesion between cells through a protein–protein molecular handshake mechanism [44]. As a prototypic single self-recognizing protein, it has proved an attractive method of generating intercellular adhesion [45]. Here, we combined adhesion with an artificial patterning system based on segregating incompatible plasmids [26,46]. This technique transforms cells with multiple plasmids that can be rendered incompatible, resulting in plasmid segregation at division. This process marks cell lineages in colonies arising from a single cell, generating distinct spatial domains in a controlled and reproducible manner [46]. The boundaries between such domains exhibit a fractal-like shape due to the uniaxial growth of the bacteria [26], which leads to the elongation of aligned cell files which successively buckle and fold. To study the spatial structure within colonies, we used microscopy and image analysis to quantify the boundaries between domains, finding that adhesion extended boundaries when both domains are adhesive, increasing the mixing and area of interaction between lineages. To study the pattern forming process dynamically, we employed time-lapse microscopy and motion tracking techniques to find that intercellular adhesion increased mixing by increasing rotational motion during colony growth. We added a model of intercellular adhesion to CellModeller, and found that adhesive interactions between cells were sufficient to reproduce the experimental results *in silico*, and demonstrated that adhesion increased the propagation of forces through the colony.

## 2. Results

### 2.1. Genetic regulation of intercellular adhesion

**Figure 1.**
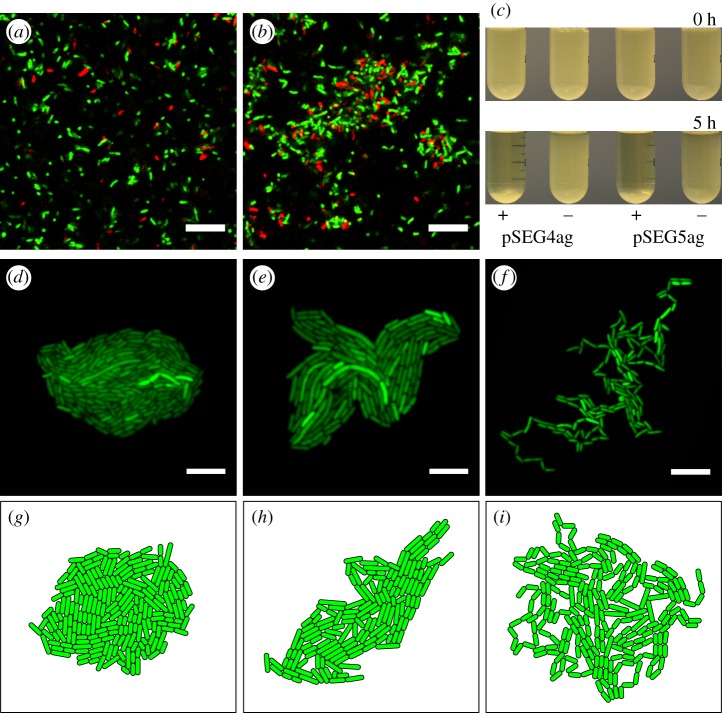
Genetic regulation of the adhesive phenotype via *ag43* expression. Cells containing accessory plasmid pL31N and either sfGFP labelled pSEG4ag or mCherry labelled pSEG5ag exhibited inducible clumping with (*b*) 1 mM IPTG unlike (*a*) without IPTG in liquid culture due to *ag43* induction. (*c*, top panel) Overnight cultures with pSEG4ag or pSEG5ag and pL31N with (+) 1 mM IPTG or (−) 0 IPTG were left to stand (bottom panel) unshaken for 5 h, displayed autoaggregation. Microcolonies grown on agar pads showed significant morphological changes with adhesion in (*d*) pSEG4ag and pL31N without IPTG, (*e*) pSEG4ag and pL31N with 1 mM IPTG, (*f*) strongly adhesive plasmid pKAYag with 100 mM arabinose. CellModeller colonies with adhesive interactions show similar colony morphology when compared with micrographs at adhesion strengths (*g*) 0, (*h*) 0.05 and (*i*) 10 in simulation units. Scale bars, 10 μm.

Intercellular adhesion was introduced with the *ag43* adhesin, cloned by PCR from the genome of *E. coli* TOP10 (Invitrogen). The *ag43* gene was expressed by the pLlac-O1 promoter [47] on plasmids pSEG4ag and pSEG5ag, which also contained constitutively expressed *sfGFP* and *mCherry* fluorescent proteins, respectively. The pLlac-O1 promoter is bound and consequently repressed by the LacI repressor protein, and this repression is lifted by the allolactose molecular mimic Isopropyl *β*-d-1-thiogalactopyranoside (IPTG), inducing transcription from the promoter. Thus, when these plasmids were combined with the accessory plasmid pL31N, containing constitutively expressed repressor LacI, cells exhibited IPTG induced autoaggregation, with cells clumping in liquid culture (figure 1*a*,*b*), and falling out of suspension (figure 1*c*), characteristic of *ag43* adhesin expression [42].

Expression of *ag43* in surface growing microcolonies grown from single cells also generated striking morphological phenotypes, with representative colonies shown in figure 1*d–f*. *Ag43* expression from pSEG4ag resulted in the slight deformation of colonies to less circular colony outlines (figure 1*e*). When expressed from a strong arabinose inducible promoter on a plasmid with higher copy number, pKAYag, colonies exhibited a ‘chaining’ phenotype, arising from cells adhering at their poles after division [48] (figure 1*f*; electronic supplementary material, figure S4). These microcolony phenotypes were however transient, as growing cell files buckled to fill the empty space between cells, returning colonies to a circular morphology (electronic supplementary material, figure S4).

Adhesion was introduced into the CellModeller software as an intercellular interaction, due to the specific nature of the Ag43 protein–protein interaction, and since no substrate binding has been reported for this protein. The adhesive forces act as a viscous drag force on cells in contact transverse to the vector normal of the contact, to counteract slippage between cells (a detailed mathematical description can be found in electronic supplementary material, figure S5 and section SI4). Adhesion strength was assumed to be uniform across the cell surface, consistent with the homogeneous surface distribution of Ag43 found in immunostaining assays [48]. Through the modulation of this adhesive force, the model was able to computationally recapitulate microcolonies with qualitatively similar morphologies as observed in experiments. At zero adhesion strength, the simulation generated microcolonies with circular morphologies (figure 1*g*); intermediate adhesion strengths produced some disruption to circularity figure 1*h*); and higher adhesion strengths produced microcolonies containing isolated chains of cells (figure 1*i*; electronic supplementary material, figure S5*g*). While these comparisons are qualitative due to the difficulty of quantifying the parameters of the bacterial system, the microcolony phenotypes in the model emerged only through the modulation of intercellular adhesion. The recapitulation of the experimentally observed morphological features, and their dependence on adhesion strength, suggested that the model had captured some of the important aspects of adhesion.

### 2.2. Artificial patterning

In order to observe the effects of adhesion on the structure of growing colonies, the inducible *ag43* cassette was expressed from plasmid backbones that formed an artificial patterning system that generated colonies with distinct spatial domains from one initial cell [46].

**Figure 2.**
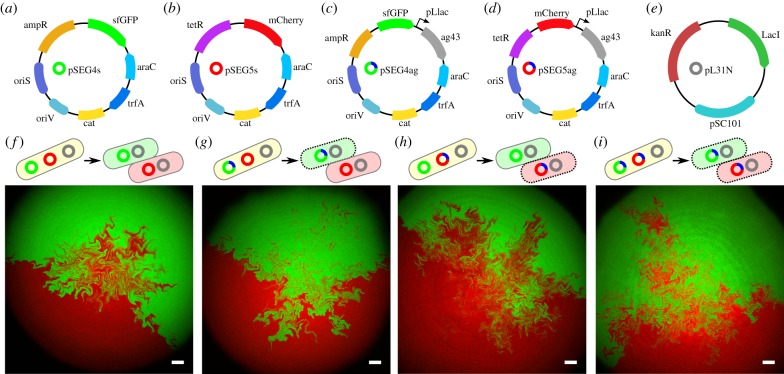
The artificial patterning system was composed of segregating plasmids (*a*) pSEG4s, (*b*) pSEG5s, (*c*) pSEG4ag, (*d*) pSEG5ag and (*e*) accessory plasmid pL31N. The segregating plasmids were controllably compatible, and were combined into single cells and made to segregate to generate two-domain colonies with variable adhesive properties: (*f*) pSEG4s and pSEG5s, (*g*) pSEG4ag and pSEG5s, (*h*) pSEG4s and pSEG5ag and (*i*) pSEG4ag and pSEG5ag. (*a*–*i*) Scale bar, 100 μm.

The segregating plasmid system was based on two plasmids, pSEG4s and pSEG5s (figure 2*a*,*b*), which each contained two origins of replication, a constitutive low copy number origin, *oriS*, and an arabinose inducible high copy number origin, *oriV* , as well as chloramphenicol resistance. Furthermore, pSEG4s contained constitutively expressed *sfGFP* fluorescent protein and an ampicillin resistance cassette, while pSEG5s contained *mCherry2* and tetracycline resistance, allowing for the maintenance of both plasmids together at high copy number with dual selection, and detection through distinct fluorescent markers. Both plasmids exhibited the expected copy number increase from induction with arabinose (electronic supplementary material, figure S2*a*,*b*), and could both be maintained in cells in the presence of arabinose, carbenicillin and tetracycine. Autoaggregation was not observed in cells containing pSEG4s and pSEG5s (electronic supplementary material, figure S3).

The segregating plasmids were augmented with a gene cassette containing *ag43* driven by the inducible pLlac-O1 promoter to create adhesive plasmids pSEG4ag and pSEG5ag (figure 2*c*,*d*). In the presence of IPTG in the growth medium, this cassette therefore induced the expression of *ag43* in the presence of the LacI repressor, which was constitutively expressed from accessory plasmid pL31N. The promoter was characterized by using a distinct fluorescent protein in the place of *ag43* to measure the IPTG response, which found promoter activation above 100 μM at all arabinose levels (electronic supplementary material, figure S2*c*,*d*).

Plasmids pSEG4ag and pSEG5ag, as well as non-adhesive pSEG4s and pSEG5s were then used to generate colonies where none, one or both of the domains were adhesive in the presence of IPTG. In order to simplify the analysis of the patterns generated, we opted to restrict our analysis to two-domain colonies, which corresponded to segregation events early during colony growth [46]. In these experiments, we transformed *E. coli* cells with either plasmid pSEG4s or pSEG4ag and either pSEG5s or pSEG5ag, as well as accessory plasmid pL31N (figure 2*f–i*). After incubation in conditions where the plasmids were compatible and at high copy number, the media was changed to render them incompatible. The resulting cultures were then incubated further to reduce the copy number of segregating plasmids and increase the frequency of two domain colonies, before being plated onto LB agar plates containing kanamycin and chloramphenicol and either 0 or 1 mM IPTG. A total of 86 two-domain colonies were imaged at high-resolution after 24 h of incubation at 37°C, with four representative examples shown in figure 2*f–i*.

### 2.3. Adhesion extends boundaries between domains

**Figure 3.**
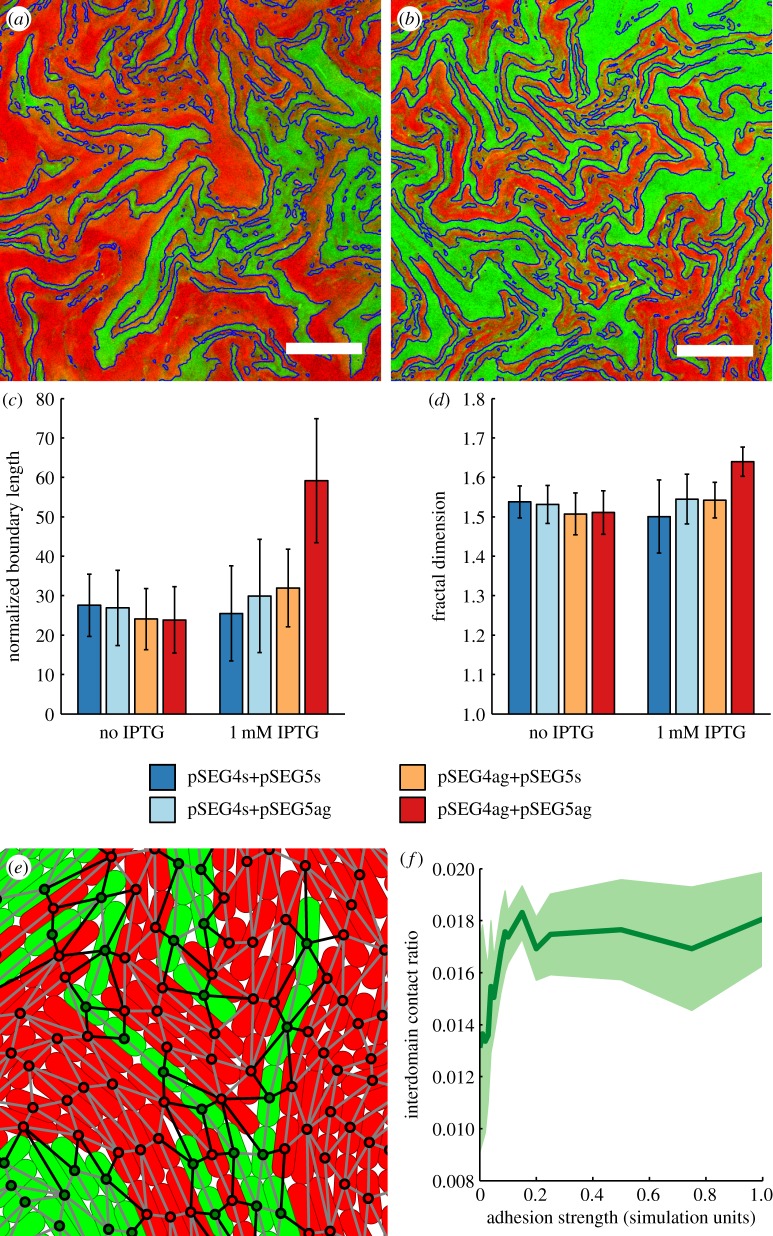
The morphological effects of adhesion on two-domain bacterial colonies. Close up example snapshots of centres of a colony with (*a*) neither domain being adhesive, pSEG4s and pSEG5s (*b*) both domains adhesive, pSEG4ag and pSEG5ag, both with 1 mM IPTG and 50 μm scale bars. The boundary extracted by the image processing algorithm is highlighted in blue. (*c*) Normalized boundary length for colonies in all conditions, (*d*) fractal dimension of the boundary object in colonies, error bars in (*c*,*d*) represent the standard deviation between sample colonies. To quantify the length of the boundary between domains computationally, (*e*) the interdomain cellular contacts (black) were compared to intradomain contacts (grey) in CellModeller four colonies of 50 000 cells. (*f*) Average ratio of interdomain contacts as a function of adhesion strength of all contacts in simulations. The data show 34 simulations averaged by adhesion strength value, and the light green region around the line represents the standard deviation.

The emergence of distinct spatial domains in colonies arising from a single cell facilitated study of the spatial structure of bacterial populations, as the context in which the domains arise was easily reproducible, unlike with studies using mixed bacterial populations. The patterns found in *ag43* expressing colonies appeared qualitatively more jagged and buckled on a smaller length scale, as shown in figure 3*a*,*b*. To quantify the effect, we measured the length of the fractal-like boundary between the two domains, as it can be reliably extracted from images. Furthermore, the boundary plays a functional role determining the interaction area between cell lineage populations, therefore providing a measure of mixing between the two populations. The boundary was extracted with an edge-detection algorithm, shown in blue in figure 3*a*,*b*, and the resulting line length was divided by the colony radius to control for colony size, leaving a dimensionless parameter. The normalized boundary length, in figure 3*c*, showed a significant increase (*p* = 1.2 × 10^−5^) when both cell types were adhesive, however no such effect was observed when only one of the cell types was adhesive. The fractal dimension of the boundary was also measured (figure 3*d*), and also showed a significant increase (*p* = 1.4 × 10^−4^), again only when both cell types were adhesive. The change in fractal dimension suggests that adhesion altered the process of pattern generation only when both domains were adhesive. Furthermore, the increase in fractal dimension of the boundary line suggested it was more space-filling, which was consistent with a longer boundary line between the domains of the colony.

To study this effect computationally, CellModeller simulations of two-domain colonies were performed to observe the boundary between domains with varying intercellular adhesion. To simulate the experimental set-up, colonies were initiated from two identical touching cells, coloured green and red, with varying adhesion strength between all cells, and simulated until colonies contained 50 000 cells. A total of 34 such simulations were performed at a wide range of values of adhesion strength. As a contiguous boundary line between domains was not well defined between individual cells in point contact, the interaction area between the lineages was measured by number of contacts between cells of different domains (shown in black in figure 3*e*) as a ratio of all intercellular contacts. The results (figure 3*f*) showed a positive dependence of the interdomain contact ratio on adhesion strength, demonstrating that adhesive physical interactions were sufficient to reproduce the experimentally observed phenomenon of boundary elongation.

### 2.4. Adhesion increases rotational motion during colony growth

**Figure 4.**
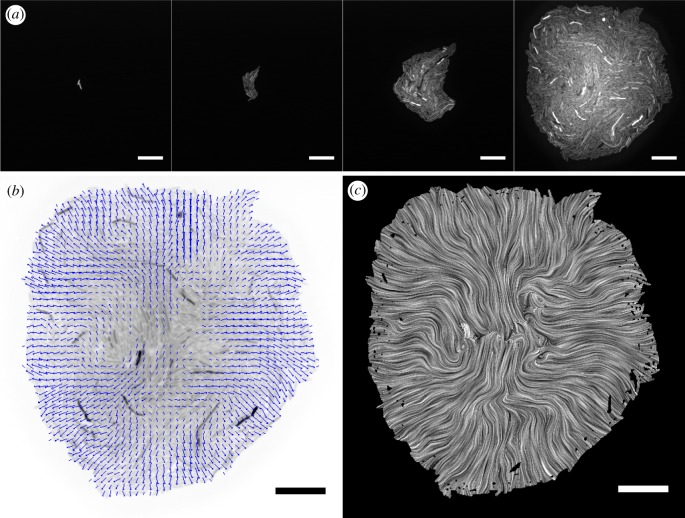
(*a*) A montage of a growing *E. coli* colony with adhesive pSEG4ag plasmid and accessory pL31N in the presence of 1 mM IPTG at frame number 1, 14, 27 and 40 corresponding to 0, 130, 260 and 390 min of the time lapse. (*b*) The velocity field between the final frame of panel (*a*) and the preceding time point, obtained by the optical flow algorithm overlaid with blue arrows, where the arrows represent the distance moved in 10 min. (*c*) The same velocity field as in (*b*), visualized over the colony with the line integral convolution (LIC) technique [50]. Scale bars, 20 μm.

In order to understand the mechanistic process underlying the boundary elongation effect from adhesion, we investigated the spatial dynamics of growth, which drives the warping and buckling of the boundary. As the boundary structure is fundamentally made of bacterial cells, we employed single-cell resolution time-lapse microscopy of adhesive and non-adhesive bacterial colonies. *E. coli* cells containing either pSEG4ag or pSEG4s and accessory plasmid pL31N were imaged in the GFP channel in a spinning disc confocal microscope every 10 min, at 37°, growing on agar pads from a few initial cells (figure 4*a*). To measure the dynamics of growth and the pattern forming process, the motion of cells was measured in 31 such growing colonies using the Farneback dense optical flow [49] algorithm to obtain the velocity fields. The optical flow algorithm was first validated and parameterized using CellModeller simulation data, where it was able to estimate the velocity fields in a growing colony with an error rate of around 2% per pixel (discussed in electronic supplementary material, section SI5).

**Figure 5.**
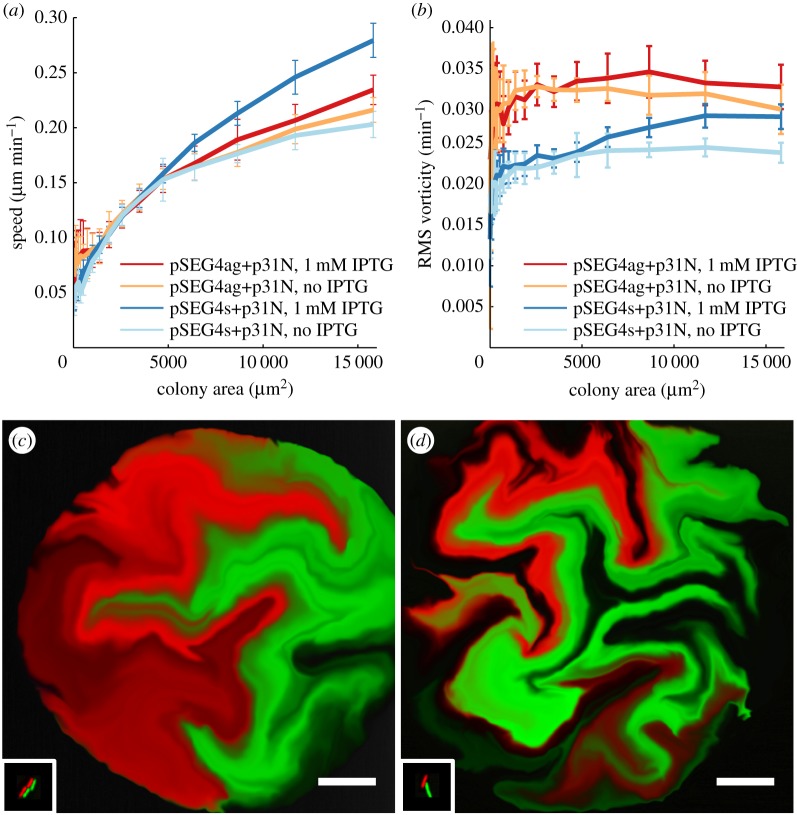
Velocity field analysis of growing bacterial colonies from time-lapse microscopy. (*a*) The speed (velocity magnitude) of the velocity field averaged over the colony as a function of colony area for each time lapse condition. (*b*) Root mean square vorticity measured across the colony area showed an increase in rotational motion in the adhesive case. Error bars in both cases represent standard deviation of the variable in sample colonies. The extracted velocity field from time lapses was successively applied to the initial frame in which cells were falsely coloured (inset in the bottom left). This process revealed the lineages of the initial cells for (*c*) a non-adhesive colony (pSEG4s+pL31N, 1 mM IPTG), and (*d*) an adhesive colony (pSEG4ag+pL31N, 1 mM IPTG), and showed increased mixing in the adhesive case adhesion. Scale bars, 20 μm.

The velocity fields of the growing colonies revealed velocity vectors radiating out from the colony centre, with increasing mean speed as the colonies grew larger (figure 5*a*). When the *ag43* gene was not present on the plasmid, the addition of IPTG increased the magnitude of the velocity field, although a similar effect was not found when *ag43* was present, presumably due to the metabolic load on growth from *ag43* expression. Visualization of the velocity field using the line integral convolution (LIC) technique [50] showed vortices and regions of rotational motion during growth (figure 4*c*). These regions of rotational motion were not fixed in space during growth, and were mostly constrained near the centre of the colonies.

Rotational motion within the colony is intimately linked to boundary elongation, as it rotates groups of cells, thus warping and extending the boundary lines between domains of cells. In order to quantify the rotational motion, and thus boundary elongation and mixing, we measured the vorticity of the velocity vector fields of the growing colonies, which represents the rotational motion per unit area. Vorticity was distributed about zero as rotations were occurring in both directions, therefore to quantify the absolute amount of rotation in a colony at a particular time interval, we calculated root mean square (RMS) vorticity. The results, in figure 5*b*, showed that colonies containing the *ag43* bearing plasmids had a higher background level of vorticity, likely from leaky basal expression leading to some *ag43* being present even without IPTG. In the presence of IPTG, colonies containing pSEG4ag displayed the highest levels of vorticity in the experiment, significantly higher than in the pSEG4s case (*p* = 6.3 times 10^−5^ at the largest colony area point). This result demonstrated that *ag43* mediated intercellular adhesion resulted in more rotational motion within growing bacterial colonies.

We also observed the spatial structure of the lineages by false colouring the initial cells of the time-lapse with distinct colours, and successively applying the extracted velocity fields to distort the initial image. Figure 5*c*,*d* shows representative images, demonstrating an increase in mixing for the adhesive case. Furthermore, this process directly demonstrated how regions of rotation serve to warp and extend the boundary between lineages. The increased rotational motion from adhesion therefore served to increase the boundary length, as observed in the large colony experiments.

**Figure 6.**
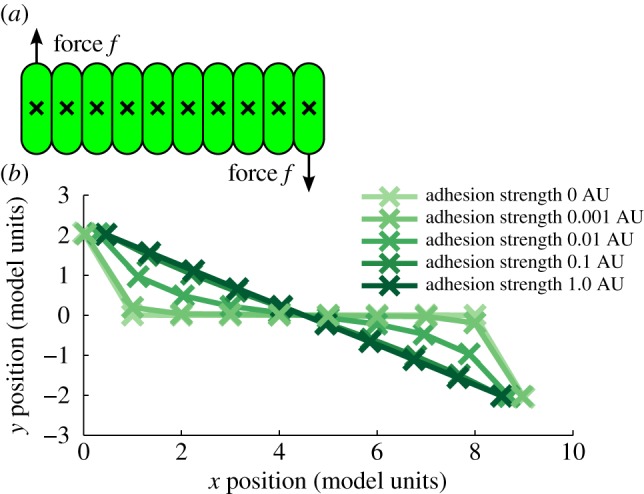
(*a*) CellModeller simulation of a chain of 10 cells in contact, with constant forces applied to the end-most cells. (*b*) The positions of all 10 cells plotted after a fixed displacement of the end cells with varying adhesion, showing that increased adhesion allows for the propagation of force and motion to neighbouring cells.

Individual bacteria within a growing bacterial colony generate forces along their axis, which are organized in locally aligned domains [51], with the many such aligned domains oriented at random to each other. The disparate orientations of these domains within the colony, and their collective forces due to growth can result in rotational motions, stirring the cells, and buckling and folding the boundaries between cell lineages. To understand how adhesion affects the motion of cells in more detail, we used a highly simplified *in silico* model. In a CellModeller simulation of a chain of 10 identical non-growing cells in contact along their long axes (figure 6*a*), we applied forces to the end-most cells of the chain and observed the displacement of all cells as a function of adhesion strength. Figure 6*b* shows the position of all cells after a fixed displacement of the end cells, showing how adhesion coupled neighbouring cells spatially. Adhesion allowed moving cells to displace their neighbours and increased adhesion allowed forces to propagate further. In the colony context, adhesion therefore serves to increase the diffusion of forces, including those responsible for rotational motion, contributing to the observed increase of vorticity.

## 3. Discussion and conclusion

During biofilm growth, cells are growing and interacting both physically and chemically, leading to the formation of emergent structure. In order to understand how intercellular adhesion impacts biofilm architecture, we have used a synthetic system to generate intercellular adhesion in specific domains within bacterial colonies. We first demonstrated regulated intercellular adhesion, which led to autoaggregation in liquid culture and had striking effects on microcolony morphology. We then combined intercellular adhesion with an artificial patterning system to generate domains with particular adhesive properties in bacterial colonies arising from single cells, which facilitated the measurement of the influence of local interactions on larger scale colony structure. Through a combination of high resolution time-lapse microscopy and modelling, we found that adhesion increased mixing by extending the boundaries between cell lineages. Stronger adhesive coupling of cells allowed forces to propagate further, and increased rotational motion which mixed the cells inside colonies and extended boundaries between lineages.

A significant feature of our experimental approach was the ease with which internal colony structure can be observed and measured through the use of an artificial patterning system. As the patterns were generated inside a single colony, initiated from a single cell, they could be created robustly and reproducibly. This facilitated the direct observation of macroscopic changes brought about by local intercellular interactions, and allowed for their quantification. The combination of these genetic tools with high-magnification time-lapse observations validated our findings and provided insight into the dynamics of the process. While the time-lapse data showed some boundary elongation, the extent of the larger scale effects would not be obvious from that data alone. Furthermore, modelling with CellModeller was complementary, allowing for computational study of the adhesive interaction and furthering the development of image processing tools. The recapitulation of the experimentally observed boundary elongation *in silico* provided further evidence that physical intercellular adhesion was sufficient for the morphological phenomena. Our findings suggest that our experimental approach can be further successfully employed to study the spatial organization resulting from a wide range of cellular behaviours and interactions. Employing both experimental and modelling techniques can be complementary for understanding dynamic mechanisms, which can often be challenging in systems with many interacting agents.

Our results indicate that simple physical interactions can have significant implications for the dynamics of colony growth and the structure of bacterial colonies. The optical flow analysis on the time-lapse data presented a novel view of colonial growth, and suggested that the mixing of bacteria in the colony is similar to the process of stirring by chaotic advection [52], whereby chaotic mixing is generated outside of turbulent regimes. In such systems, blinking or moving [53] vortices can result in chaotic mixing and fractal formation in low Reynolds number regimes without conventional turbulence.

Non-motile rod-shaped bacteria in colonies exhibit local nematic ordering within domains of aligned cells [26], and so can be viewed from the perspective of liquid crystals. In this context, the bacteria constitute an active liquid crystal system, due to the extension and division of individual cells. Such active liquid crystal systems display many unusual non-equilibrium phenomena, and their study is a rapidly emerging field [54]. One relevant feature of such active liquid crystal systems is active turbulence, whereby the collective behaviour of particles results in spontaneous chaotic motions [55]. In extensile liquid crystal systems, where the constituent particles of the liquid crystal extend over time, defects in alignment can occur spontaneously, and act as sources of motion and vorticity, producing active turbulence [56]. Our work highlights that the collective organization occurring in bacterial colonies is a similar class of active turbulence phenomenon. Growing bacteria within a colony generate defects in nematic order through buckling instability [51], and these defects act as sources of vorticity which serve to create the fractal-like boundary patterns between lineages [26]. Furthermore, our work has demonstrated how engineered intercellular interaction can modulate this process.

As simple local interactions are relatively easy to engineer, understanding and exploiting their emergent effects can radically simplify large generating morphological and phenotypic changes in multicellular structure. Our work has developed the methods to study the spatial effects of physical mechanisms which, combined with models, can be exploited to design and control the spatial structure of microbial populations. Furthermore, we have used these methods to characterize a tool for the spatial bioengineering of microbial communities. The *ag43* adhesin can be used to increase the interaction area between different members, which could improve the efficiency of processes involving consortia members which are exchanging metabolites and signals in engineered microbial systems.

## 4. Methods

### 4.1. Plasmids

All plasmids were built using PCR and Gibson assembly [57] (see electronic supplementary material, Methods section for more details). Descriptions of the plasmids used throughout this work are shown in more detail in electronic supplementary material, S1.

### 4.2. Bacterial culture

For the autoaggregation experiments, liquid LB media with appropriate antibiotics was inoculated with transformed *E. coli* TOP10 and grown for 16 h at 37°C in a shaking incubator. The overnight cultures were diluted to an identical optical density, then left to stand in a rack at room temperature, then photographed.

For the colony segregation experiments, 5 ml liquid LB media containing kanamycin (50 μg ml^−1^), tetracycline (10 μg ml^−1^), carbenicillin (100 μg ml^−1^) and 100 mM arabinose was inoculated with *E. coli* TOP10 transformed with pSEG4s or pSEG4ag and pSEG5s or pSEG5ag plasmids, and the accessory plasmid pL31N. After 5 h of growth in a 37°C shaking incubator, the cultures were centrifuged at 3000*g* at 20°C for 10 min, and the supernatant replaced with LB media containing only chloramphenicol (12 μg ml^−1^) and kanamycin (50 μg ml^−1^). After a further 3 h of 37°C shaking incubation, the OD was measured, and the culture was diluted such that 100 μl would contain 100 cfu, which were plated onto 9 mm plates containing LB and 1.5% (w/v) Bactoagar with kanamycin (50 μg ml^−1^) and chloramphenicol (12 μg ml^−1^) and either 0 or 1 mM IPTG. Eighty-six two-domain colonies were then imaged in the GFP (excited at 488 nm, detected at 490–510 nm) and RFP channels (excited at 561 nm, detected at 605–653 nm), corresponding to a sample size of approximately 11 for each of the eight experimental conditions. We imaged in a confocal microscope after 24 h of growth on plates in a static 37°C incubator. Further microscopy details can be found in electronic supplementary material, methods.

For the time-lapse microscopy experiments, transformed *E. coli* TOP10 cells were grown on a small pad (approx. 1 cm × 1 cm × 1 mm) of LB and 1.5% Bactoagar and antibiotics, that was covered by a glass coverslip and enclosed by a microscope slide and several GeneFrames (ThermoScientific). Sample preparation and microscopy is covered in greater detail in electronic supplementary material, Methods. Microscopy was performed in a 37°C incubated confocal microscope, imaging the GFP channel at several positions on the agar pad every 10 min.

### 4.3. Data analysis

Custom python image analysis scripts were used to process the data. For large colony image analysis, the two channel images (representing sfGFP and mCherry domains) were smoothed and thresholded to yield binary images for each channel. To measure the boundary between cell lineages, edges of the binary objects were found. In order to remove the outline of the colony, the boundary of the two binary channels combined was subtracted from the boundary in each channel. The resulting boundaries were then skeletonized to form a line of single pixel width, and subsequently the length of that line was found by summing the number of pixels. To control for colony size, the boundary length was normalized by colony radius. Fractal dimension was found by using the Euclidean distance mapping method [58].

The Farneback dense optical flow algorithm [49] was initially validated using simulation data from CellModeller with a known velocity field, where simulations had the same pixelation, length scale and growth time scale as the time-lapse microscopy data (see electronic supplementary material, section S5 for further detail). An optimal parameter set was found by minimizing the error of the optical flow derived velocity field from the known simulation velocity field (electronic supplementary material, figure S7), and these parameters were used in all further analysis. In order to calculate vorticity, the curl of the resulting velocity field was found, since vorticity was distributed about zero, the RMS vorticity was found and averaged across the colony area at each time point. Colonies from separate time lapses were compared at the equivalent colony area.

Statistical analysis was performed using a two-sample equal variance Student’s *t*-test.

### 4.4. Modelling

Cellular modelling was performed with using the CellModeller 4.3 [41] software on an Hewlett Packard workstation running Ubuntu v. 14.3. During this work, intercellular adhesion was implemented in the CellModeller, and the code be found on the ‘adhesion’ branch of the CellModeller github repository. For a detailed description of mathematics and implementation of adhesion, please see electronic supplementary material, section S4.

## Supplementary Material

Supplementary Information

## Data accessibility

CellModeller software is open-source and freely available through http://haselofflab.github.io/CellModeller/. All imaging scripts developed as part of this work, and representative imaging data are available at https://github.com/antonkan/Imaging-Scripts.
