# Supplementary material for: Intercellular adhesion promotes clonal mixing in growing bacterial populations
Source: J R Soc Interface. 2018 Sep 19;15(146):20180406. doi: 10.1098/rsif.2018.0406 (PMC6170782; doi:10.1098/rsif.2018.0406)
Supplement: Supplementary Information [file rsif20180406supp1.pdf]

Intercellular adhesion promotes clonal mixing in growing bacterial populations

Supplementary information

Anton Kan[1†], Ilenne Del Valle[2], Tim Rudge[4,5], Fernán Federici[2,3], and Jim Haseloff[1,*]

[1]Department of Plant Sciences, University of Cambridge, Cambridge, UK
[2]Departamento de Genética Molecular y Microbiologa, Facultad de Ciencias Biológicas, Pontificia Universidad Católica de Chile, Santiago, Chile
[3]Fondo de Desarrollo de Áreas Prioritarias, Center for Genome Regulation, Millennium Institute for Integrative Systems and Synthetic Biology (MIISSB), Santiago, Chile
[4]Institute for Biological and Medical Engineering, Schools of Engineering, Biology and Medicine, Pontificia Universidad Católica de Chile, Santiago, Chile
[5]Department of Chemical and Bioprocess Engineering, School of Engineering, Pontificia Universidad Católica de Chile, Santiago, Chile
[*]To whom correspondence should be addressed. E-mail: jh295@cam.ac.uk
[†]Current address: Wyss Institute for Biologically Inspired Engineering, Boston, 02115, USA. Email: anton.kan@wyss.harvard.edu

Supplemental methods

Plasmid construction All plasmids were built from fragments amplified by PCR with *Phusion*® High-Fidelity DNA Polymerase (NEB), using custom synthesized oligonucleotides (Sigma Aldrich). Amplified fragments were extracted from an agarose (Sigma Aldrich) gel. Linear DNA fragments were purified using the MinElute PCR purification kit (QIAGEN), and assembled into plasmids with Gibson Assembly [1]. *E. coli* TOP10 (Invitrogen) was used throughout cloning and further experiments. Plasmids were purified from overnight cultures using the QIAprep spin miniprep kit (QIAGEN). Plasmid were verified by Sanger sequencing from Source Bioscience. All plasmids are shown in more detail in section SI1.

Time-lapse sample preparation A small agar pad was made by pouring molten LB (Sigma Aldrich) and 1.5% agar (Bactoagar, BD Biosciences), supplemented with chloramphenicol ($12\mu g/mL$) and kanamycin ($50\mu g/mL$), into a chamber made of a single GeneFrame (1.5cm×1.6cm, ThermoScientific) stuck onto a microscope slide, which was then covered by a glass cover slip and allowed to set. The coverslip was then removed carefully, and the agar pad was inoculated with bacteria. The pad was then cut into a square of ∼1cm×1cm and placed bacteria side down onto a clean cover slip, and stuck onto a stack of 3 GeneFrames affixed to a microscope slide, such that the agar pad was on the cover slip, inside the chamber of GeneFrames and glass, and surrounded on the remaining 3 sides with air.

To prepare the bacteria sample, *E. coli* TOP10 cells, containing either plasmids pSEG4s or pSEG4ag and accessory plasmid pL31N, were grown to exponential phase in liquid LB media with chloramphenicol ($50\mu g/mL$) and kanamycin ($50\mu g/mL$), and at 37°C in a shaking incubator. During mid-exponential phase (OD=0.1-0.4) the cells were diluted such that $1\mu L$ would contain ∼5-50 *cfu*, and $1\mu L$ was applied onto the LB-agar pad, and allowed to dry for several minutes at 37°C.

Imaging Macro photography was performed using a Canon EOS 550D SLR

Large two-domain colony images were taken on a Leica SP5 confocal microscope after 24 hours of colony growth. A 10x air objective was used (HCX PL APO CS 10.0x0.40 DRY), and sfGFP was excited at 488nm and emission detected 490-510nm, mCherry2 at 561nm and detected at 605-653nm. Colonies were dome shaped, around 1.5mm in diameter and on average around 110μm tall at the centre, falling to single cell thickness at the edges. The colonies were thus imaged as a stack of ∼40 horizontal confocal slices, which were combined into a single image for analysis by taking a maximum intensity projection.

Time-lapse movies were taken on a custom Nikon Eclipse Ti inverted microscope with a Yokogawa CSU-X1 spinning disk module, hardware autofocus with Nikon Perfect Focus and an enclosed incubator (Okolab) set to 37°C. Images were captured with a Photometrics Evolve (512x512 EMCCD) camera, with image acquisition performed using the Metamorph software (Molecular Devices). Fluorescence images of the cell layer closest to the coverslip were taken with a 60x objective (CFI Plan Apo VC 60X Oil) at multiple positions to track many colonies, every 10 minutes for 16 hours. sfGFP excited at 488nm, and emission detected at 525nm (ET525/50m filter).

1 Plasmids

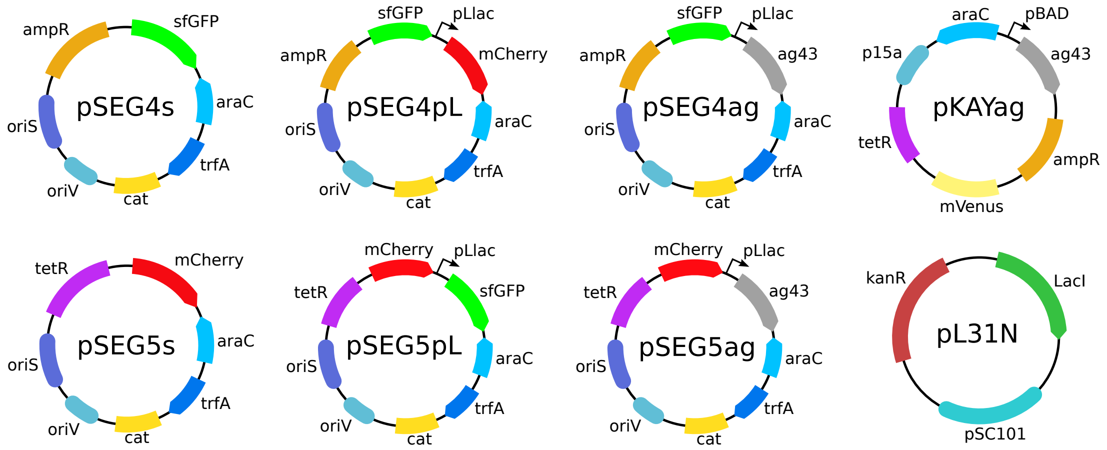

Figure S1: Schematics of all plasmids used throughout this study.

Table 1: Diagrams of all the plasmids used, showing the plasmid backbone and additional transcriptional units, described by protein and some promoter names.

Plasmid name	Description	Source
pKAYag	*pSB4K5, ampR, araC, pBAD:ag43, J23101:eYFP*	This study
pSEG4s	*pETcoco-1, ampR, J23101:sfGFP*	This study
pSEG5s	*pETcoco-1, tetR, J23101:mCherry*	This study
pSEG4pL	*pETcoco-1, ampR, J23101:sfGFP, pLlac:mCherry*	This study
pSEG5pL	*pETcoco-1, tetR, J23101:mCherry, pLlac:sfGFP*	This study
pSEG4ag	*pETcoco-1, ampR, J23101:sfGFP, pLlac:ag43*	This study
pSEG5ag	*pETcoco-1, tetR, J23101:mCherry, pLlac:ag43*	This study
pL31N	*pSB4K5, J23101:lacI*	This study

Plasmids and fully annotated sequences shown in fig. S1 and table 1 will be available on Addgene.

Plasmid characterization

The plasmid segregation mechanism relied on the segregation plasmids being able to increase their copy number in response to arabinose. This was characterized for plasmids pSEG4s and pSEG5s using plate fluorometry and plasmid DNA quantification, and shown in figure S2a and b. Both plasmids showed a large increase in DNA yield and fluorescence intensity at arabinose levels above $\sim 0.1\ mM$, demonstrating the required increase in copy number.

Plasmids pSEG4s and pSEG5s were augmented with a transcriptional unit containing *ag43* driven by *LacI* repressed pLlac promoter [2] to create plasmids pSEG4ag and pSEG5ag. This promoter was characterized using plasmids pSEG4pL and pSEG5pL, which contained the pLlac promoter driving a spectrally distinct fluorescent protein. Fluorescence was measured in cultures containing pSEG4pL or pSEG5pL, with *LacI* bearing accessory plasmid

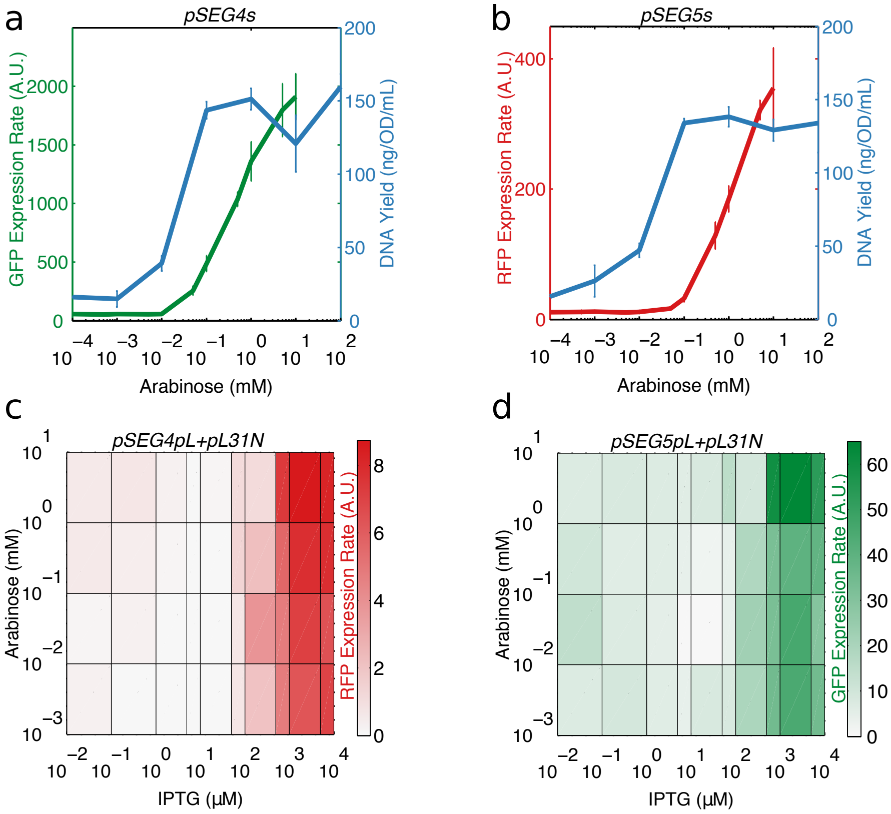

Figure S2: Plasmid characterization. (a) pSEG4s and (b) pSEG5s increase in copy number and the fluorescent marker expression as a function of arabinose concentration. These plasmids were further augmented for characterization by a cassette with pLlac-O1 promoter driving mCherry2 to make (c) pSEG4pL and driving sfGFP in (d) pSEG5pL, which exhibited IPTG induction in gene expression when combined with accessory plasmid pL31N. Error bars represent standard deviation between triplicate data.

pL31N. The results (fig. S2c-d) showed high promoter activity at IPTG levels above 100 μM at all arabinose levels.

1.1 Materials and methods

Plate Fluorometry Samples were prepared for plate fluorometry by diluting overnight cultures 1:1000 into fresh media containing the appropriate antibiotics, with the LB media used throughout. The resulting inoculated media was loaded into a black 96-well, flat clear bottom plate (Grenier). If an inducer was used, 5 L of the inducer was added to each well at the appropriate concentration with 195 L of sample, otherwise 200 L of inoculated media was used. As a control, each plate also contained at least 3 wells containing LB media and *E. coli* TOP10 cells without plasmids.

The places were then loaded into in a BMG Clariostar plate reader, where they were grown in LB with the appropriate antibiotics at 37°C. Fluorescence and absorbance readings

were taken every 10 minutes for 16 hours, with shaking between readings. The following settings were used on the CLARIOstar machine: sfGFP: excitation 470/15 nm, emission 515/20 nm, gain 800, mCherry: excitation 570/15 nm, emission 620/20 nm, gain 1100, OD: 600 nm.

Data was subsequently analysed in the MATLAB (MathWorks) software. Background autofluorescence in each channel was removed by subtracting the fluorescence intensity of the control wells (with blank *E. coli* cells) at the appropriate OD. Fluorescent protein expression rates were defined as the fluorescence intensity gradient per OD [(dF/dt)/OD], averaged across an exponential phase window (OD = 0.2 - 1.0).

DNA quantification Plasmid DNA quantification was performed by purifying plasmid DNA from overnight cultures, incubated at 37°C in LB media with chloramphenicol ($10\mu g/\mu L$) and kanamycin ($50\mu g/\mu L$), and varying levels of arabinose. Minipreparations were made using the QIAprep spin miniprep kit (QIAGEN), and DNA concentration was measured using a NanoVue (GE Healthcare Life Sciences).

2 Autoaggregation in liquid culture

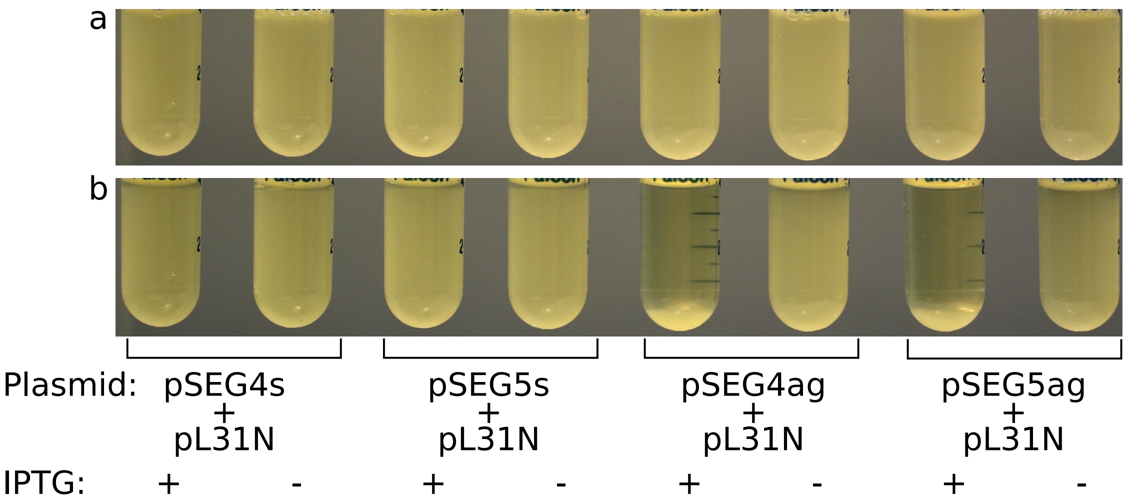

Figure S3: (a) *E. coli TOP10* liquid cultures grown overnight in LB and a shaking incubator at 37°C, containing the pL31N accessory plasmid and one of following plasmids plasmids: pSEG4s, pSEG5s, pSEG4ag, pSEG5ag (indicated in the table below). Each plasmid combination was grown with either 100mM IPTG (+) or 0 IPTG (-). (b) The same cultures as in panel A after 5 hours standing at room temperature, showing that IPTG induced expression of *ag43* led to cellular autoaggregation to the bottom of the flask.

3 Adhesive colony morphotypes

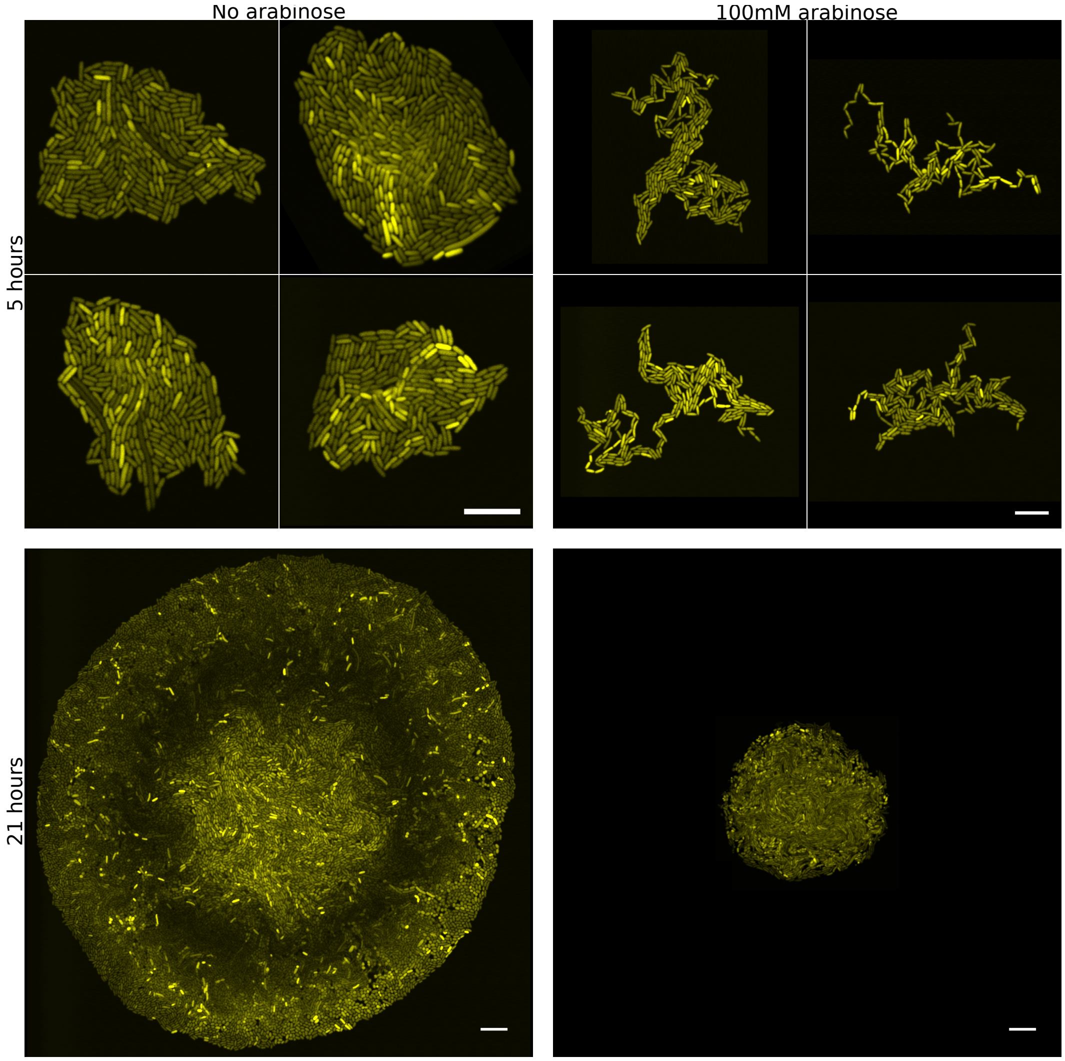

Figure S4: Confocal micrographs of *E. coli* TOP10 cells with the *pPBag43* plasmid, containing the pBAD promoter driving the *ag43* adhesin. Colonies were grown at 37°C on M9 agar pads at 0 and 100mM arabinose. Unusual morphology can be seen in colonies imaged at 5 hours, however, this morphology was not found in colonies imaged after 21 hours. Scale is 10 μm.

3.1 Materials and methods

Microcolonies in figure S4 were grown on 1.5% agar (Bactoagar, BD Biosciences) pads and M9 (Ameresco) media supplemented with 0.4% (w/v) L-glucose (Riedel-de Haen) and 0.2%

(w/v) casamino acids (Fisher Scientific). Bacterial cultures were grown to exponential phase, diluted and placed onto the M9 agar pad, covered by a glass coverslip and enclosed in a chamber made of several 1.5cmx1.6cm GeneFrames (Thermo Fisher Scientific) and a glass microscope slide. Slides were incubated in a static 37°C incubator and imaged in a Leica SP5 confocal microscope using a 40x objective (HCX PL APO CS 40.0x1.45 OIL UV) and with the following fluorescence settings: excitation 514 nm, emission 521-555 nm.

4 CellModeller computation adhesion model

4.1 CellModeller basics

A full derivation of the mathematics underpinning CellModeller can be found in [3, 4], as well as in the CellModeller documentation in `https://github.com/HaseloffLab/CellModeller/blob/master/Doc/Maths/math.pdf`. However a brief introduction will be presented here as background to adhesive interactions.

A cell in CellModeller is modelled by a capsule with 7 coordinates $\tilde{\mathbf{x}} = (\mathbf{x}\ \theta\ l)^T$, with $\mathbf{x}$ representing the location of the cell centre in 3-dimensional space, θ the 3-dimensional orientation of the cell, and l the 1-dimensional cell length. Forces in the simulation are provided by cell growth, and cells feel a viscous drag force proportional to their velocity. To understand a cell's motion from a generalized force, we can study the cellular change in coordinates given a general impulse $\Delta\tilde{\mathbf{p}}$, represented by $(\Delta\mathbf{p}\ \Delta\mathbf{L}\ \Delta g)^T$, with linear, angular and growth components. A change in cell of initial length l_0 coordinates can then be give by:

$$\Delta\tilde{\mathbf{x}} = \begin{bmatrix} \frac{\Delta\mathbf{p}}{\mu l_0} \\ \frac{12\mathbf{P}\Delta\mathbf{L}}{\mu l_0^3} \\ \frac{\Delta g}{\gamma} \end{bmatrix} \tag{1}$$

$$= \begin{bmatrix} \frac{1}{\mu l_0} & 0 & 0 \\ 0 & \frac{12\mathbf{P}}{\mu l_0^3} & 0 \\ 0 & 0 & \frac{1}{\gamma} \end{bmatrix} \begin{bmatrix} \Delta\mathbf{p} \\ \Delta\mathbf{L} \\ \Delta g \end{bmatrix} = \mathbf{M}^{-1}\Delta\tilde{\mathbf{p}} \tag{2}$$

Matrix $\mathbf{M}$ therefore determines the change in motion from the impulse, with a term for viscous drag on translational motion, rotational motion, and growth. μ represents the drag coefficient to motion, whereas γ presents the drag coefficient with respect to growth. The matrix $\mathbf{P}$ is the projection onto the plane perpendicular to the cell axis. A full derivation can be found in the references above.

For any surface point $\mathbf{q}$ on a cell with a center at $\mathbf{q_a}$, direction $\hat{\mathbf{a}}$ and length l_0, we can find the displacement of $\mathbf{q}$ generated by $\Delta\tilde{\mathbf{x}}$ along any axis $\hat{\mathbf{h}}$ by calculating:

$$\Delta q_{\hat{h}} = \begin{bmatrix} \hat{\mathbf{h}}^T & -(\hat{\mathbf{h}} \times \mathbf{q})^T & \frac{(\hat{\mathbf{h}}\cdot\hat{\mathbf{a}})(\mathbf{q}\cdot\hat{\mathbf{a}})}{l_0} \end{bmatrix} \Delta\tilde{\mathbf{x}} = \mathbf{B}\mathbf{M}^{-1}\Delta\tilde{\mathbf{p}} \tag{3}$$

4.2 Intercellular Adhesion

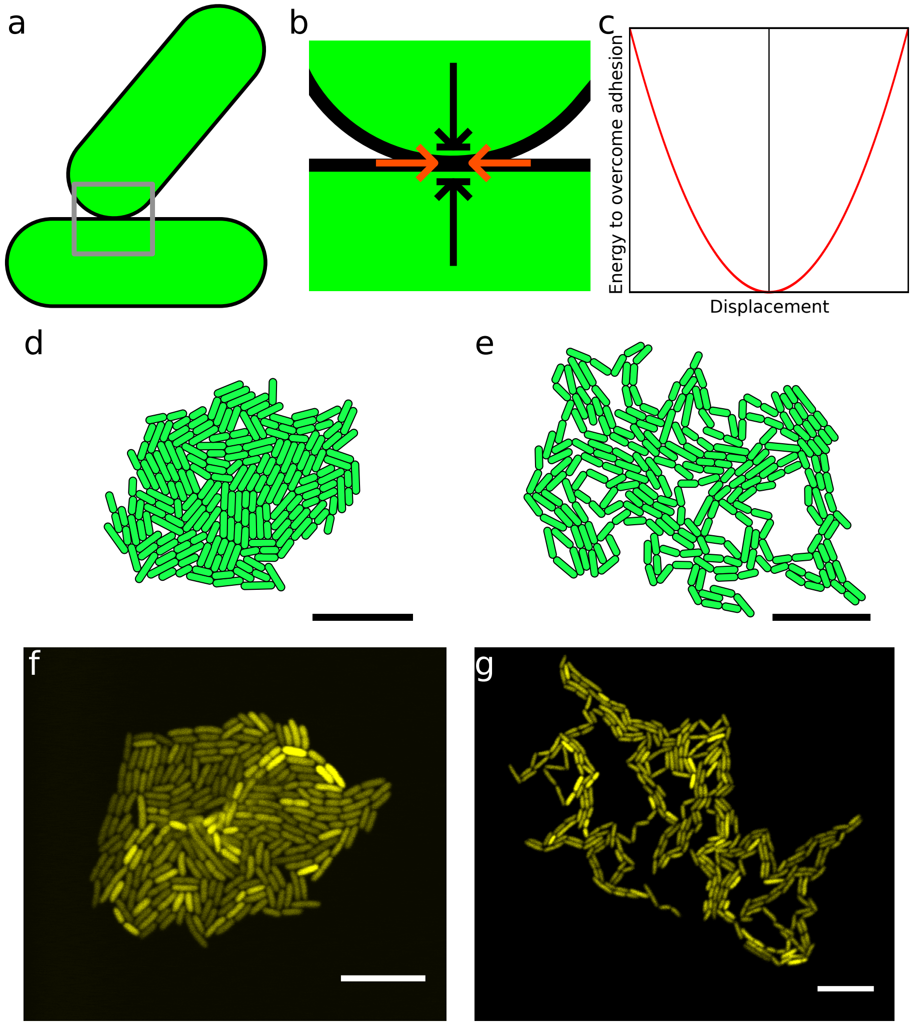

Figure S5: Modelling intercellular adhesion in CellModeller. (a) For two cells in contact in the simulation, (b) adhesion was modelled as force opposing transverse motion cells about the contact (red arrows). (c) This sets up a parabolic potential well at the contact point, with motion away from the original point of contact penalized energetically by adhesion. This model generated similar colony morphologies to experimentally found ones: simulated colonies with around 200 cells and (d) 0 adhesion strength and (e) an adhesion strength parameter set to 10, appeared similar to *E. coli* microcolonies with *pPBag43* grown for 5 hours on an M9 agar pad with either (f) 0 arabinose and (g) 100 mM arabinose. Scale bars are 10 m.

Adhesion is modelled as a simple linear elastic spring between the contact points, where the restoring force is proportional to the displacement between the cell contact points (fig. S5a-b).

The bacterial biophysics in CellModeller calculates the impulses on cells such that the distance between any two touching cells is 0 in the axis normal to the contact. Therefore to model adhesion between cells in contact, we only need to consider displacement in the plane orthogonal to the normal.

The energy stored in an elastic spring is given by $E = \kappa x^2$ where κ is the elastic constant of the spring, and represents the adhesion strength of the contact. This linear spring therefore produces a parabolic potential well, with the energy proportional to the square of the displacement (fig. S5c). Minimizing this energy at each timestep constrains the cells to move with adhesion between contact points.

In a system of two cells A and B, which have a contact, indexed as i, at points $\mathbf{p_a}$ and $\mathbf{p_b}$ on each of the cells. Impulses on the two cells generate a displacement between two contact points on the cells. The displacement, Δd_f, generated in an axis tangential to the contact, $\hat{\mathbf{f}}$, can be found in terms of impulses using equation 3, giving:

$$\begin{aligned} -\Delta d_{fi} &= \begin{bmatrix} \mathbf{d}_{ai}\mathbf{M}_a^{-1} & -\mathbf{d}_{bi}\mathbf{M}_b^{-1} \end{bmatrix} \begin{bmatrix} \Delta\mathbf{p}_a \\ \Delta\mathbf{p}_b \end{bmatrix} && (4) \\ &= \mathbf{G}_i \Delta\mathbf{p} && (5) \end{aligned}$$

Therefore, the energy stored in adhesion at contact i is given by:

$$\begin{aligned} E_i &= \kappa_i \Delta d_{fi}^2 && (6) \\ &= \kappa_i (\mathbf{G}_i \Delta\mathbf{p})^2 && (7) \\ &= (\Delta\mathbf{p}\mathbf{G}_i)^T \kappa_i \mathbf{G}_i \Delta\mathbf{p} && (8) \end{aligned}$$

The simulation solves all impulses such cell overlaps at contacts, defined by $\mathbf{d}$, are minimized. This is done by solving $\mathbf{A}\Delta\mathbf{p} = -\mathbf{d}$, where $\mathbf{A}$ is a block matrix with $n_{contacts} \times n_{cells}$, and represents the system of cellular contacts and the associated axes of each contact with relation to a cell. This acts to project each impulse for a given cell in the appropriate axis, thus defining how an impulse affects each contact overlap.

Typically, $\mathbf{A}$ is poorly conditioned, meaning that there are many solutions. This therefore requires regularization, which is performed with Tikhunov regularization [5], which finds the solution which minimizes the energy. The adhesive energy can be added into the Tikhunov regularization term, which constrains the solution towards the solutions that minimize the work done against drag and adhesive energies. Therefore, the expression that needs to be minimized by the solver is given by:

$$\begin{aligned} \epsilon &= \min_{\Delta p}\{||\mathbf{A}\Delta\mathbf{p} + \mathbf{d}||^2 + \alpha(E_{drag} + E_{adh})\} && (9) \\ &= \min_{\Delta p}\{||\mathbf{A}\Delta\mathbf{p} + \mathbf{d}||^2 + \alpha(\mathbf{M}(\mathbf{M}^{-1}\Delta\mathbf{p})^2 + (\Delta\mathbf{p}\mathbf{G})^T \kappa \mathbf{G}\Delta\mathbf{p})\} && (10) \end{aligned}$$

To minimize this expression, we find the derivative of ϵ with respect to $\Delta\mathbf{p}$.

$$\frac{d\epsilon}{d(\Delta\mathbf{p})} = 0 = \mathbf{A}^T(\mathbf{A}\Delta\mathbf{p} + d) + \alpha(\mathbf{M}^{-1}\Delta\mathbf{p} + \mathbf{G}^T\kappa\mathbf{G}\Delta\mathbf{p}) \quad (11)$$

$$(\mathbf{A}^T\mathbf{A} + \alpha(\mathbf{M}^{-1} + \mathbf{G}^T\kappa\mathbf{G}))\Delta\mathbf{p} = -\mathbf{A}^T\mathbf{d} \quad (12)$$

This expression therefore minimizes the adhesive energy with respect to tangential displacement in 1 axis. In the 2D case, therefore, it is required only once, as the tangential axis $\hat{\mathbf{f}}$, is always perpendicular to the contact and the $\hat{\mathbf{z}}$ axis (assuming the cells are constrained in the $\hat{\mathbf{x}} - \hat{\mathbf{y}}$ plane). In the more general 3 dimensional case, two axes in the plane tangential to the contact are defined on the fly, and the adhesion energies in each axis are calculated and added and minimized.

4.3 Implementation

The implementation of this model of intercellular adhesion can be found on `http://haseloafflab.github.io/CellModeller/`, within on the 'Adhesion' branch of the GitHub repository.

Within the biophysical model, the term κ defined the strength of the adhesive interaction for a given contact. For implementation, the adhesion strength between each cell type can be set in the initialization of each cell. The logic of adhesion can also be defined by the user in the model in the `adhLogicCL` function, which is parsed into the openCL code. This function sets how different cell types with differing adhesion strengths interact. For example, the following code:

```
def adhLogicCL():
        """return min(adh_str1, adh_str2);"""
```

defines the adhesion strength of a contact by the minimal adhesion strength of the cells in contact. However this can be altered in a variety of ways to model a wide range of adhesive behaviour.

A usage guide for the adhesion module can be found in the documentation on the CellModeller website.

4.4 Usage notes

At intermediate adhesion the spatial packing of cells was less efficient than in experiments. This was likely due to the interaction between the agar pad and the colony which serve to compress the cells in the colony together [6], which was not explicitly accounted for in the model. However, these forces are only present when the bacteria are between an agar pads and glass cover slips, and are not present in colonies growing on an agar surface.

5 Optical flow optimization

The Farneback optical flow algorithm [7], used in chapter 6 on time-lapse microscopy data was first parameterized and validated with simulation data. To do this, CellModeller simulations required the appropriate length and timescales, to demonstrate that the optical flow finds the appropriate motion, and to find parameters optimized for motion in the time-lapse microscopy data.

Therefore, colonies were simulated growing from a single cell, saving an snapshot of the simulation with a frequency such that area growth of the simulation matched experimental data (fig. S6c). Furthermore, the resolution of the snapshots was set to match experimental data (512x512 pixels), and the width of the field of view also matched the data (136.5 μm). The image sequence of the simulated colonies (fig. S6b) therefore appeared similar to experimental data (fig. S6a). Furthermore, the velocity field between each frame of the simulation was found, and exported to a file.

The optical flow algorithm, implemented in python using the `openCV` package [8], requires several parameters which are listed below, along with the descriptions from the python openCV documentation (`http://docs.opencv.org/2.4/modules/video/doc/motion_analysis_and_object_tracking.html`):

- **pyr_scale** parameter, specifying the image scale (< 1) to build pyramids for each image; pyr_scale=0.5 means a classical pyramid, where each next layer is twice smaller than the previous one.

- **levels** number of pyramid layers including the initial image; levels=1 means that no extra layers are created and only the original images are used.

- **winsize** averaging window size; larger values increase the algorithm robustness to image noise and give more chances for fast motion detection, but yield more blurred motion field. iterations number of iterations the algorithm does at each pyramid level.

- **poly_n** size of the pixel neighborhood used to find polynomial expansion in each pixel; larger values mean that the image will be approximated with smoother surfaces, yielding more robust algorithm and more blurred motion field, typically poly_n =5 or 7.

- **poly_sigma** standard deviation of the Gaussian that is used to smooth derivatives used as a basis for the polynomial expansion; for poly_n=5, you can set poly_sigma=1.1, for poly_n=7, a good value would be poly_sigma=1.5.

- **flags** operation flags that can be a combination of the following:

  OPTFLOW_USE_INITIAL_FLOW uses the input flow as an initial flow approximation.

  OPTFLOW_FARNEBACK_GAUSSIAN uses the Gaussian winsize x winsize filter instead of a box filter of the same size for optical flow estimation; usually, this option gives z more accurate flow than with a box filter, at the cost of lower speed; normally, winsize for a Gaussian window should be set to a larger value to achieve the same level of robustness.

In order to find a parameter set that would allow for the Optical Flow algorithm to accurately find the velocity fields in experimental data, an exhaustive parameter search

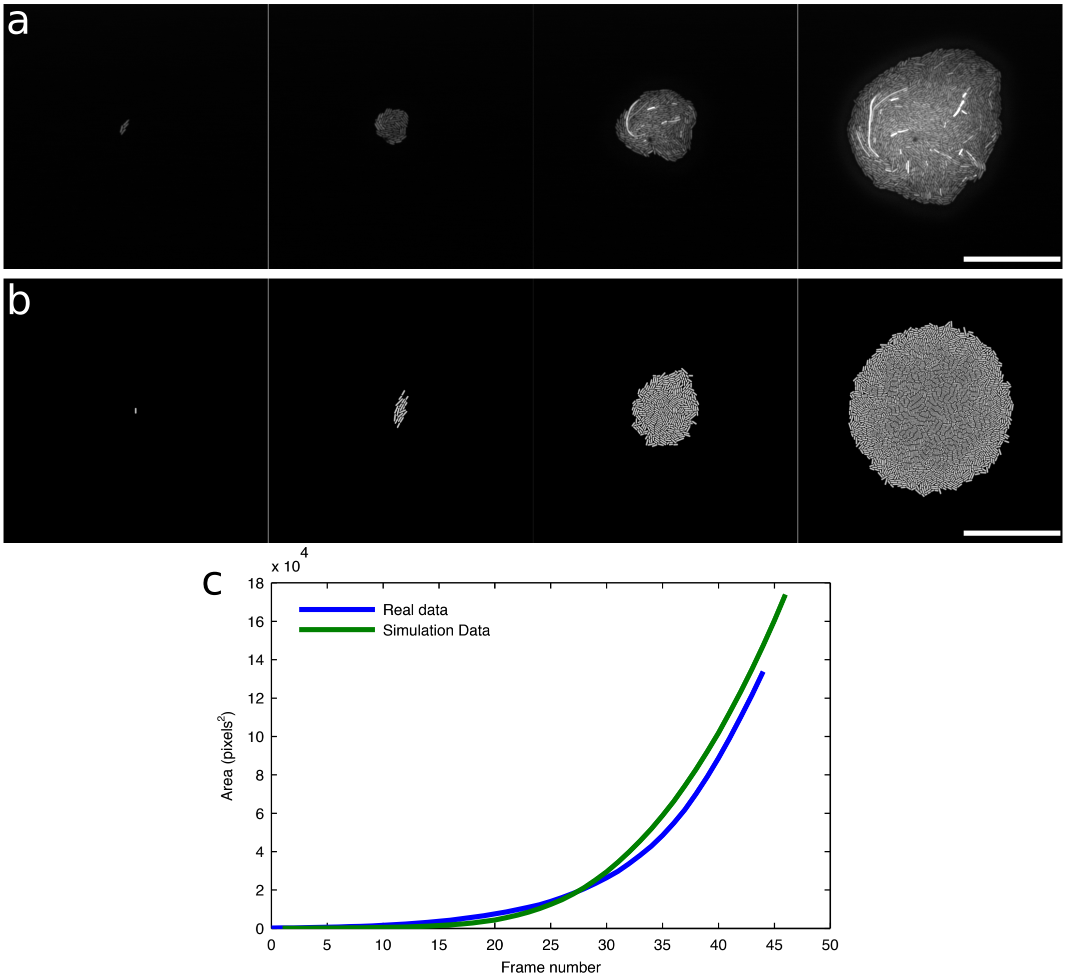

Figure S6: (a) Montage of time-lapse microscopy data of *E. coli* TOP10 cells with plasmids pSEG4s and accessory plasmid pL31N, growing on an M9 agar pad with 100 mM IPTG, chloramphenicol and kanamycin, showing a growing colony at frame 0, 12, 24 and 36, corresponding to 0, 120, 240 and 360 minutes. (b) CellModeller simulation data, shown at the same frame number as the time-lapse, with the an equivalent image pixelation and length scale. (c) CellModeller growth rate was set such that the area growth between frames matched the microscopy data. Scale bars are 50 μm.

was performed using a simulated dataset with known velocity fields. The simulations were designed to be of equivalent image pixelation (512x512), length scale (cell width = 0.75 μm, cell length = 1 - 4 μm) and time scale between frames (figure S6c). A total of three such simulations were performed and used for parameter optimization.

To calculate the error of the optical flow output, the optical flow derived velocity field, F_{opt} was subtracted from the exact velocity field provided by the CellModeller simulation, F_{sim}. The resulting magnitude of this vector was then found for each pixel, and then summed in quadrature with values from other pixels containing cells. To calculate the error

per pixel as a percentage, the total error was normalized by the magnitude of the real velocity fields, to obtain the following expression for the error per pixel:

$$Error\ per\ pixel = \frac{\sqrt{\sum_{colony}(|F_{sim} - F_{opt}|)^2}}{\sum_{colony}|F_{sim}|} \tag{13}$$

Iteration over all parameters was performed one parameter at a time. Once a minimum was found for the first parameter, the value was saved and iteration proceeded over the next parameter, and so on. This was performed several times to find a global minimum. Iterations about the final parameter set used are shown in figure S7a, finding a final parameter set with an error rate per pixel of around 2%. The final parameter set accurately recapitulated the model velocity field well, as shown in figure S7c and d.

The final parameter set chosen was the following:

- **pyr_scale** =0.25
- **levels** = 3
- **winsize** = 25
- **poly_n** = 5 (Note that this was a slightly suboptimal choice, however, the documentation recommended that this be kept at either 5 or 7)
- **poly_sigma** = 0.7
- **flags** = 256 (OPTFLOW_USE_INITIAL_FLOW) (This flag was used throughout since the velocity field at any given point was similar to the previous time point)

Therefore, the code calling the optical flow algorithm is as follows:

```
cv2.calcOpticalFlowFarneback(prev, img, None,
                             0.5, 3, 25, 28, 5, 0.7, 256)
```

Where `prev` refers to the initial or previous image of the image sequence, and `img` the subsequent image.

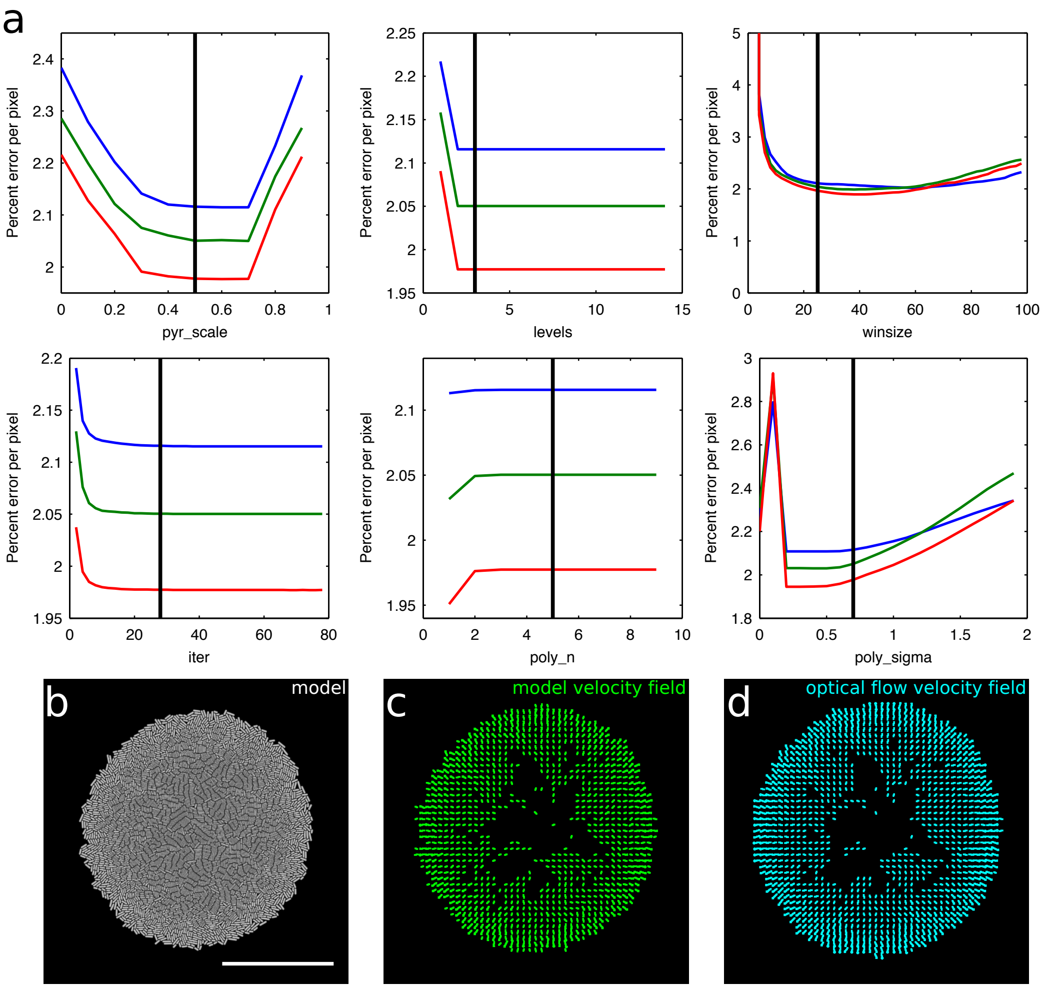

Figure S7: (a) Exhaustive parameter iteration across all 6 parameters of the optical flow algorithm for 3 separate simulation datasets, shown in red, green and blue. The y-axis in each case shows the percentage error per pixel. The vertical bold line in each plot represents the final parameter value used. When the optical flow algorithm uses the final parameter set on simulation data video, it accurately recapitulates the velocity field of the simulation. Panel (b) shows a snapshot of simulation data at the 40th frame, and (c) shows the velocity field as a quiver plot for the frame calculated by the simulation software, almost indistinguishable from the optical flow derived field in (d). Arrow lengths represent the distance travelled every 10 minutes. Scale bars are 50 μm.

References

[1] D. G. Gibson, L. Young, R.-Y. Chuang, J. C. Venter, C. A. Hutchison, and H. O. Smith, "Enzymatic assembly of DNA molecules up to several hundred kilobases," *Nature Methods*, vol. 6, pp. 343–345, May 2009.

[2] R. Lutz and H. Bujard, "Independent and Tight Regulation of Transcriptional Units in Escherichia Coli Via the LacR/O, the TetR/O and AraC/I1-I2 Regulatory Elements," *Nucleic Acids Research*, vol. 25, pp. 1203–1210, Mar. 1997.

[3] T. J. Rudge, P. J. Steiner, A. Phillips, and J. Haseloff, "Computational modeling of synthetic microbial biofilms," *ACS Synthetic Biology*, vol. 1, no. 8, pp. 345–352, 2012.

[4] T. J. Rudge, "Phd thesis," 2015.

[5] A. Neubauer, "Tikhonov regularisation for non-linear ill-posed problems: optimal convergence rates and finite-dimensional approximation," *Inverse Problems*, vol. 5, no. 4, p. 541, 1989.

[6] M. A. A. Grant, B. Wacaw, R. J. Allen, and P. Cicuta, "The role of mechanical forces in the planar-to-bulk transition in growing Escherichia coli microcolonies," *Journal of The Royal Society Interface*, vol. 11, p. 20140400, Aug. 2014.

[7] G. Farnebck, "Two-Frame Motion Estimation Based on Polynomial Expansion," in *Image Analysis* (J. Bigun and T. Gustavsson, eds.), no. 2749 in Lecture Notes in Computer Science, pp. 363–370, Springer Berlin Heidelberg, June 2003.

[8] G. Bradski and others, "The opencv library," 2000.
